# [(Dibenzo[*b*,*d*]thio­phen-4-yl)tellan­yl]methane­thiol

**DOI:** 10.1107/S160053681100273X

**Published:** 2011-01-29

**Authors:** Zuo-Qin Liang, Xu-Tang Tao

**Affiliations:** a State Key Laboratory of Crystal Materials, Shandong University, Jinan 250100, People’s Republic of China

## Abstract

In the title compound, C_13_H_10_S_2_Te, the dibenzothio­phene moiety is almost planar, the maximum atomic deviation being 0.055 (5) Å. The two Te—C bonds are nearly perpen­dicular to each other with a C—Te—C bond angle of 93.0 (2)°. An inter­molecular C—H⋯π inter­action is present between the methyl­ene group and thio­phene ring.

## Related literature

For general background to the field-effect transistors of organotellurium derivatives, see: Inokuchi *et al.* (1987 ▶). For related structures, see: Kobayashi *et al.* (2005 ▶).
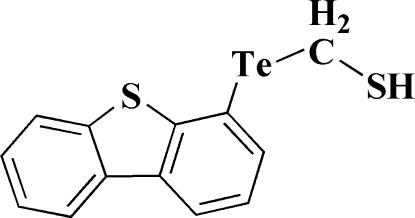


## Experimental

### 

#### Crystal data

C_13_H_10_S_2_Te
*M* *_r_* = 357.93
Orthorhombic, 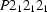

*a* = 5.49518 (12) Å
*b* = 12.1422 (3) Å
*c* = 19.0529 (5) Å
*V* = 1271.27 (6) Å^3^
*Z* = 4
Mo *K*α radiation
μ = 2.64 mm^−1^
*T* = 293 K
0.58 × 0.45 × 0.31 mm

#### Data collection

Oxford Diffraction Xcalibur Eos Gemini diffractometer
Absorption correction: multi-scan (*CrysAlis PRO RED*; Oxford Diffraction, 2009 ▶) *T*_min_ = 0.310, *T*_max_ = 0.495
5117 measured reflections
2509 independent reflections
2248 reflections with *I* > 2σ(*I*)
*R*_int_ = 0.054

#### Refinement

*R*[*F* ^2^ > 2σ(*F* ^2^)] = 0.034
*wR*(*F* ^2^) = 0.083
*S* = 0.98
2509 reflections
146 parameters
H-atom parameters constrained
Δρ_max_ = 0.81 e Å^−3^
Δρ_min_ = −0.55 e Å^−3^
Absolute structure: Flack (1983 ▶), 975 Friedel pairs
Flack parameter: 0.01 (3)

### 

Data collection: *CrysAlis PRO CCD* (Oxford Diffraction, 2009 ▶); cell refinement: *CrysAlis PRO CCD*; data reduction: *CrysAlis PRO RED* (Oxford Diffraction, 2009 ▶); program(s) used to solve structure: *SHELXTL* (Sheldrick, 2008 ▶); program(s) used to refine structure: *SHELXTL*; molecular graphics: *SHELXTL*; software used to prepare material for publication: *SHELXTL*.

## Supplementary Material

Crystal structure: contains datablocks global, I. DOI: 10.1107/S160053681100273X/xu5144sup1.cif

Structure factors: contains datablocks I. DOI: 10.1107/S160053681100273X/xu5144Isup2.hkl

Additional supplementary materials:  crystallographic information; 3D view; checkCIF report

## Figures and Tables

**Table 1:** Hydrogen-bond geometry (Å, °) *Cg* is the centroid of the thio­phene ring.

*D*—H⋯*A*	*D*—H	H⋯*A*	*D*⋯*A*	*D*—H⋯*A*
C13—H13*A*⋯*Cg*^i^	0.97	2.90	3.846 (6)	166

Symmetry code: (i) 
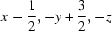
.

**Fig. 1.**
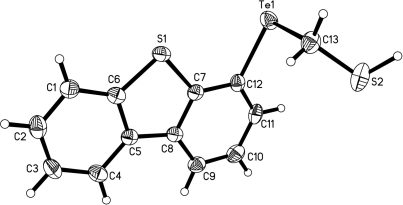
The molecular structure of (I), showing 30% probability displacement ellipsoids and the atom-numbering scheme.

**Fig. 2.**
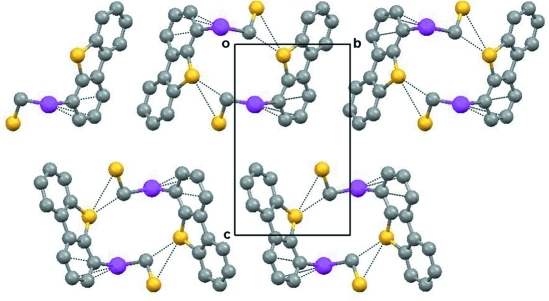
Stacking diagram of (I), viewed down the a axis, showing intermolecular Te···π, C···S and S···S interactions (dashed lines). The hydrogen atoms have been omitted for clarity.

## supplementary crystallographic information

### Comment

The design and synthesis of high-performance organic semiconductor materials have been an active topic in the area of field-effect transistors. Recently, organotellurium compounds have received significant attention due to the high charge carrier mobility (Inokuchi *et al.*, 1987). In this paper, we report the synthesis and the crystal structure of the title compound (I).

The asymmetric unit of the title compound is shown in Fig. 1. The dibenzothiophene group possesses perfect planarity: the dihedral angle between the two phenyls is 4.11°. The two Te—C bonds are nearly perpendicular to each other with a C—Te—C bond angle of 93.0 (2)°. In the packing structure (Fig. 2), the molecules are packed into molecular columns along the *a* axis through intermolecular Te···π interactions between the tellurium atom and the phenyl ring (C7—C12). The contact distance of Te···C9 is 3.68 (5) Å, that of Te···C10 is 3.47 (5) Å, and that of Te···C11 is 3.73 (7) Å. These contact distances are significantly shorter than the sum of the van der Waals radii of tellurium and aromatic carbon atoms (Kobayashi *et al.*, 2005). The molecular columns are connected together by intermolecular C···*S* and *S*···S interactions. The contact distances of C13···S1 and S1···S2 are 3.43 (5) Å and 3.51 (2) Å, respectively, which are obviously shorter than the sum of the corresponding van der Waals radii. There are no classic hydrogen bonds in the crystal structure.

### Experimental

Addition of a 1.6 *M* solution of n-BuLi in hexane (8.50 ml, 13.6 mmol) to the THF solution of dibenzothiophene (2.50 g, 13.6 mmol) at room temperature under an Ar atmosphere. The reaction mixture was stirred for 2 h, and then tellurium powder (1.70 g, 13.3 mmol) was added. After 3 h, the reaction mixture was poured into a beaker containing 200 ml cold distilled water and oxidized by passing oxygen at a moderate rate for 1 h. The organic solvent was evaporated, and the suspension was extracted with dichloromethane. The organic extracts were washed by brine, and dried by anhydrous CaCl_2_. After the solvent was removed, the crude product was chromatographed on silica gel using petroleum as eluent to give the title compound (0.27 g, yield 5.56%). Single crystals suitable for X-ray diffraction were obtained by very slow evaporation of a chloroform/ethanol solution.

### Refinement

H atoms bonded to C atoms were fixed geometrically and allowed to ride on their attached atoms, with C—H = 0.93–0.97 Å and with *U*_iso_(H) = 1.2*U*_eq_(C). H atom bonded to S atom was refined using a rotating model, with S—H = 1.20 Å and with *U*_iso_(H) = 1.2*U*_eq_(S).

### Crystal data

**Table:**

C_13_H_10_S_2_Te	*D*_x_ = 1.870 Mg m^−^^3^
*M**_r_* = 357.93	Mo *K*α radiation, λ = 0.7107 Å
Orthorhombic, *P*2_1_2_1_2_1_	Cell parameters from 3403 reflections
*a* = 5.49518 (12) Å	θ = 3.5–28.8°
*b* = 12.1422 (3) Å	µ = 2.64 mm^−^^1^
*c* = 19.0529 (5) Å	*T* = 293 K
*V* = 1271.27 (6) Å^3^	Block, colorless
*Z* = 4	0.58 × 0.45 × 0.31 mm
*F*(000) = 688	

### Data collection

**Table:**

Oxford Diffraction Xcalibur Eos Gemini diffractometer	2509 independent reflections
Radiation source: Enhance (Mo) X-ray Source	2248 reflections with *I* > 2σ(*I*)
graphite	*R*_int_ = 0.054
Detector resolution: 16.0355 pixels mm^-1^	θ_max_ = 26.4°, θ_min_ = 3.5°
ω scans	*h* = −6→7
Absorption correction: multi-scan (*CrysAlis PRO* RED; Oxford Diffraction, 2009)	*k* = −15→7
*T*_min_ = 0.310, *T*_max_ = 0.495	*l* = −21→24
5117 measured reflections	

### Refinement

**Table:**

Refinement on *F*^2^	Secondary atom site location: difference Fourier map
Least-squares matrix: full	Hydrogen site location: inferred from neighbouring sites
*R*[*F*^2^ > 2σ(*F*^2^)] = 0.034	H-atom parameters constrained
*wR*(*F*^2^) = 0.083	*w* = 1/[σ^2^(*F*_o_^2^) + (0.0502*P*)^2^] where *P* = (*F*_o_^2^ + 2*F*_c_^2^)/3
*S* = 0.98	(Δ/σ)_max_ < 0.001
2509 reflections	Δρ_max_ = 0.81 e Å^−^^3^
146 parameters	Δρ_min_ = −0.55 e Å^−^^3^
0 restraints	Absolute structure: Flack (1983), 975 Friedel pairs
Primary atom site location: structure-invariant direct methods	Flack parameter: 0.01 (3)

### Special details

**Table:**

Geometry. All e.s.d.'s (except the e.s.d. in the dihedral angle between two l.s. planes) are estimated using the full covariance matrix. The cell e.s.d.'s are taken into account individually in the estimation of e.s.d.'s in distances, angles and torsion angles; correlations between e.s.d.'s in cell parameters are only used when they are defined by crystal symmetry. An approximate (isotropic) treatment of cell e.s.d.'s is used for estimating e.s.d.'s involving l.s. planes.
Refinement. Refinement of *F*^2^ against ALL reflections. The weighted *R*-factor *wR* and goodness of fit *S* are based on *F*^2^, conventional *R*-factors *R* are based on *F*, with *F* set to zero for negative *F*^2^. The threshold expression of *F*^2^ > σ(*F*^2^) is used only for calculating *R*-factors(gt) *etc*. and is not relevant to the choice of reflections for refinement. *R*-factors based on *F*^2^ are statistically about twice as large as those based on *F*, and *R*- factors based on ALL data will be even larger.

### Fractional atomic coordinates and isotropic or equivalent isotropic displacement parameters (Å^2^)

**Table:**

	*x*	*y*	*z*	*U*_iso_*/*U*_eq_	
Te1	0.65007 (6)	0.83287 (3)	0.120133 (19)	0.05366 (13)	
S1	0.8260 (2)	0.97156 (10)	−0.03248 (6)	0.0466 (3)	
S2	1.0630 (4)	0.65872 (16)	0.17855 (9)	0.0778 (5)	
H2	0.9995	0.5698	0.1991	0.093*	
C12	0.9348 (8)	0.9486 (4)	0.1106 (2)	0.0401 (10)	
C8	1.1849 (8)	1.0679 (3)	0.0346 (2)	0.0373 (9)	
C4	1.3695 (10)	1.1778 (4)	−0.0677 (3)	0.0486 (11)	
H4	1.4968	1.2065	−0.0412	0.058*	
C9	1.3274 (10)	1.0953 (4)	0.0922 (3)	0.0466 (11)	
H9	1.4611	1.1415	0.0868	0.056*	
C2	1.1594 (11)	1.1620 (5)	−0.1780 (3)	0.0551 (12)	
H2A	1.1483	1.1809	−0.2252	0.066*	
C13	0.8876 (11)	0.6962 (4)	0.1038 (3)	0.0570 (15)	
H13A	0.7910	0.6332	0.0895	0.068*	
H13B	0.9975	0.7137	0.0656	0.068*	
C3	1.3448 (11)	1.2056 (4)	−0.1376 (3)	0.0545 (12)	
H3	1.4548	1.2543	−0.1579	0.065*	
C6	1.0149 (8)	1.0623 (4)	−0.0788 (2)	0.0394 (10)	
C7	0.9883 (8)	0.9950 (4)	0.0445 (2)	0.0381 (10)	
C11	1.0743 (9)	0.9798 (4)	0.1662 (3)	0.0471 (12)	
H11	1.0404	0.9519	0.2105	0.057*	
C1	0.9912 (9)	1.0912 (4)	−0.1496 (3)	0.0486 (12)	
H1	0.8648	1.0631	−0.1767	0.058*	
C10	1.2698 (10)	1.0539 (5)	0.1574 (3)	0.0534 (13)	
H10	1.3611	1.0750	0.1962	0.064*	
C5	1.2002 (7)	1.1060 (4)	−0.0370 (2)	0.0380 (10)	

### Atomic displacement parameters (Å^2^)

**Table:**

	*U*^11^	*U*^22^	*U*^33^	*U*^12^	*U*^13^	*U*^23^
Te1	0.04305 (17)	0.05162 (19)	0.0663 (2)	0.00382 (16)	0.01498 (15)	0.01196 (17)
S1	0.0380 (5)	0.0542 (7)	0.0475 (6)	−0.0110 (6)	−0.0028 (5)	0.0017 (5)
S2	0.1032 (13)	0.0683 (10)	0.0621 (9)	0.0296 (10)	−0.0107 (8)	0.0080 (8)
C12	0.041 (2)	0.036 (2)	0.042 (3)	0.0116 (18)	0.006 (2)	0.004 (2)
C8	0.035 (2)	0.030 (2)	0.048 (2)	0.0031 (18)	0.001 (2)	−0.0034 (18)
C4	0.047 (3)	0.041 (2)	0.058 (3)	−0.010 (3)	0.003 (2)	−0.003 (2)
C9	0.044 (2)	0.042 (2)	0.054 (3)	−0.003 (2)	−0.008 (2)	−0.004 (2)
C2	0.066 (3)	0.055 (3)	0.045 (2)	0.011 (4)	0.010 (3)	0.006 (2)
C13	0.067 (4)	0.043 (3)	0.061 (3)	0.009 (2)	−0.006 (3)	−0.005 (2)
C3	0.058 (3)	0.045 (3)	0.060 (3)	−0.006 (3)	0.013 (3)	0.010 (2)
C6	0.036 (2)	0.036 (2)	0.046 (3)	0.0048 (19)	0.008 (2)	−0.007 (2)
C7	0.034 (2)	0.029 (2)	0.052 (3)	0.0093 (17)	0.001 (2)	−0.001 (2)
C11	0.060 (3)	0.040 (3)	0.041 (2)	0.018 (2)	0.000 (2)	0.004 (2)
C1	0.049 (3)	0.047 (3)	0.050 (3)	−0.004 (2)	−0.003 (2)	−0.001 (2)
C10	0.057 (3)	0.052 (3)	0.051 (3)	0.004 (3)	−0.015 (2)	−0.010 (2)
C5	0.036 (2)	0.033 (2)	0.045 (2)	0.0048 (17)	0.0011 (19)	−0.0028 (19)

### Geometric parameters (Å, °)

**Table:**

Te1—C12	2.111 (5)	C9—H9	0.9300
Te1—C13	2.134 (5)	C9—C10	1.376 (8)
S1—C6	1.753 (5)	C2—H2A	0.9300
S1—C7	1.739 (5)	C2—C3	1.382 (8)
S2—H2	1.2000	C2—C1	1.374 (8)
S2—C13	1.778 (6)	C13—H13A	0.9700
C12—C7	1.412 (7)	C13—H13B	0.9700
C12—C11	1.360 (7)	C3—H3	0.9300
C8—C9	1.389 (6)	C6—C1	1.399 (7)
C8—C7	1.409 (6)	C6—C5	1.397 (6)
C8—C5	1.443 (6)	C11—H11	0.9300
C4—H4	0.9300	C11—C10	1.411 (8)
C4—C3	1.381 (8)	C1—H1	0.9300
C4—C5	1.403 (7)	C10—H10	0.9300
			
Te1—C13—H13A	108.6	H13A—C13—H13B	107.6
Te1—C13—H13B	108.6	C3—C4—H4	120.4
S2—C13—Te1	114.5 (3)	C3—C4—C5	119.3 (5)
S2—C13—H13A	108.6	C3—C2—H2A	119.4
S2—C13—H13B	108.6	C6—C1—H1	120.9
C12—Te1—C13	93.0 (2)	C6—C5—C8	112.0 (4)
C12—C7—S1	125.5 (3)	C6—C5—C4	118.8 (4)
C12—C11—H11	119.5	C7—S1—C6	91.0 (2)
C12—C11—C10	121.0 (5)	C7—C12—Te1	119.8 (3)
C8—C9—H9	120.1	C7—C8—C5	111.9 (4)
C8—C7—S1	112.5 (3)	C11—C12—Te1	122.4 (4)
C8—C7—C12	122.0 (4)	C11—C12—C7	117.8 (4)
C4—C3—C2	121.0 (5)	C11—C10—H10	119.5
C4—C3—H3	119.5	C1—C2—H2A	119.4
C4—C5—C8	129.2 (4)	C1—C2—C3	121.1 (5)
C9—C8—C7	118.5 (4)	C1—C6—S1	126.0 (4)
C9—C8—C5	129.6 (4)	C10—C9—C8	119.7 (5)
C9—C10—C11	121.0 (5)	C10—C9—H9	120.1
C9—C10—H10	119.5	C10—C11—H11	119.5
C2—C3—H3	119.5	C5—C4—H4	120.4
C2—C1—C6	118.3 (5)	C5—C6—S1	112.5 (4)
C2—C1—H1	120.9	C5—C6—C1	121.5 (5)
C13—S2—H2	109.5		
			
Te1—C12—C7—S1	4.0 (5)	C6—S1—C7—C12	178.1 (4)
Te1—C12—C7—C8	−176.4 (3)	C6—S1—C7—C8	−1.5 (3)
Te1—C12—C11—C10	177.3 (4)	C7—S1—C6—C1	−177.9 (4)
S1—C6—C1—C2	−178.6 (4)	C7—S1—C6—C5	1.9 (3)
S1—C6—C5—C8	−1.9 (5)	C7—C12—C11—C10	−1.6 (7)
S1—C6—C5—C4	178.4 (4)	C7—C8—C9—C10	−1.9 (7)
C12—Te1—C13—S2	78.3 (3)	C7—C8—C5—C4	−179.5 (5)
C12—C11—C10—C9	−1.0 (8)	C7—C8—C5—C6	0.7 (5)
C8—C9—C10—C11	2.9 (8)	C11—C12—C7—S1	−177.0 (3)
C9—C8—C7—S1	178.9 (3)	C11—C12—C7—C8	2.5 (6)
C9—C8—C7—C12	−0.7 (6)	C1—C2—C3—C4	1.1 (8)
C9—C8—C5—C4	2.6 (8)	C1—C6—C5—C8	178.0 (4)
C9—C8—C5—C6	−177.2 (4)	C1—C6—C5—C4	−1.8 (7)
C13—Te1—C12—C7	91.8 (4)	C5—C8—C9—C10	175.8 (5)
C13—Te1—C12—C11	−87.1 (4)	C5—C8—C7—S1	0.7 (5)
C3—C4—C5—C8	−178.2 (5)	C5—C8—C7—C12	−178.9 (4)
C3—C4—C5—C6	1.6 (7)	C5—C4—C3—C2	−1.3 (8)
C3—C2—C1—C6	−1.2 (8)	C5—C6—C1—C2	1.6 (7)

### Hydrogen-bond geometry (Å, °)

**Table:**

Cg is the centroid of the thiophene ring.

**Table:**

*D*—H···*A*	*D*—H	H···*A*	*D*···*A*	*D*—H···*A*
C13—H13A···Cg^i^	0.97	2.90	3.846 (6)	166

Symmetry codes: (i) *x*−1/2, −*y*+3/2, −*z*.
